# Neural effects of hand-grip-activity induced fatigue sensation on appetite: a magnetoencephalography study

**DOI:** 10.1038/s41598-019-47580-z

**Published:** 2019-07-30

**Authors:** Takashi Matsuo, Akira Ishii, Chika Nakamura, Rika Ishida, Takahiro Yamaguchi, Katsuko Takada, Masato Uji, Takahiro Yoshikawa

**Affiliations:** Department of Sports Medicine, Osaka City University Graduate School of Medicine, 1-4-3 Asahimachi, Abeno-ku, Osaka, 545-8585 Japan

**Keywords:** Physiology, Cognitive neuroscience, Neuroscience

## Abstract

It has been reported that physical activity not only increases energy expenditure, but also affects appetite. However, little remains known about the effects of physical activity-induced fatigue sensation on appetite. In the present study, classical conditioning related to fatigue sensation was used to dissociate fatigue sensation from physical activity. The participants were 20 healthy male volunteers. After overnight fasting, on day 1, the participants performed hand-grip task trials for 10 min with listening to a sound. The next day, they viewed food images with (target task) and without (control task) listening to the sound identical to that used on day 1, and their neural activity during the tasks were recorded using magnetoencephalography. The subjective levels of appetite and fatigue sensation were assessed using a visual analog scale. The subjective level of fatigue increased and that of appetite for fatty foods showed a tendency toward increase in the target task while the subjective level of fatigue and that of appetite for fatty foods were not altered in the control task. In the target task, the decrease of theta (4–8 Hz) band power in the supplementary motor area (SMA), which was observed in the control task, was suppressed, and the suppression was positively correlated with appetite for fatty foods, suggesting hand grip activity-induced fatigue sensation may increase the appetite for fatty food; this increase could be related to neural activity in the SMA. These findings are expected to contribute to the understanding of the neural mechanisms of appetite in relation to fatigue.

## Introduction

The prevalence of obesity in modern society is increasing^1^. Overweight and obesity are related to increased risk for type 2 diabetes, dyslipidemia, hypertension, cardiovascular disease, and other health problems^2–6^. Body weight is determined by the balance between energy expenditure and food intake, so increasing energy expenditure and decreasing energy intake are the fundamental approaches to prevent obesity.

The level of physical activity is thought to be one of the factors that determines energy expenditure. In fact, it has been reported that obesity is associated with low physical activity^7^, and that physical activity increases energy expenditure during that activity^8,9^. In addition to the effects of physical activity on the increase in energy expenditure, there have also been reports that physical activity suppresses appetite, which has been referred to as exercise-induced anorexia^10^. For example, it has been reported that exercise on a bicycle ergometer at 70% of maximum oxygen uptake level suppressed hunger during and after the activity^10^, that exercise on a treadmill at 46% of maximum oxygen uptake level for 2 h reduced hunger and increased fullness^11^, and that swimming for 60 min suppressed appetite during the exercise^12^. Considering these findings, it has been proposed that exercise may be beneficial for those who desire to reduce body weight by enhancing the satiating effect of a meal^13^.

One of the mechanisms that suppress appetite during and after physical activity involves the effect of appetite-regulating hormones^13^. Exercise suppresses the perception of appetite and increases the amount of appetite-suppressing hormones. It has been reported that cycling exercise for 60 min at 65% maximal heart rate induces increases in plasma levels of peptide YY (PYY) and glucagon-like peptide-1 (GLP-1) and suppresses hunger in healthy normal-weight individuals^14^, and that cycling exercise for 60 min at 50% of maximum oxygen uptake level increases PYY and GLP-1 plasma levels in obese young male volunteers^15^. Alternatively, there may be factors other than the alteration of appetite-regulating hormone levels that affect appetite during and/or after physical activity, such as alterations in body temperature^16^ and gastric motility^17^.

A sensation of fatigue caused by physical activity may be one such factor that can affect appetite. Fatigue is defined as a decline in the ability to perform, or in the efficiency of performing, mental and/or physical activities as a result of excessive mental or physical activity or disease. Fatigue sensation is defined as a peculiar sense of discomfort, a desire to rest, and a decline in motivation accompanying fatigue^18^. However, to the best of our knowledge, there have been no reports investigating the effect of fatigue sensation induced by physical activity on appetite. Therefore, the aims of the present study were to clarify the effect of fatigue sensation induced by physical activity on appetite and to examine the neural mechanisms related to the alteration in appetite caused by fatigue sensation.

In order to assess the effect of fatigue sensation induced by physical activity on appetite, it is essential to dissociate fatigue sensation from physical activity because the levels of appetite-regulating hormones, body temperature, and so on can also be altered during physical activity. There have been reports that fatigue sensation can be classically conditioned. The sounds associated with fatigue sensation caused by physical activity (i.e., hand-grip trials for 10 min) and mental activity (i.e., 2-back task for 30 min) have been shown to induce fatigue sensation, even when the activities are not performed^19,20^. In the present study, we used this conditioning procedure using hand-grip trials for 10 min to induce fatigue sensation without physical activity to assess the alteration of appetite caused by fatigue sensation. Although the intensity of the physical activity in our present study (i.e., hand-grip-activity) seems to be low compared with those used in the previous studies assessed the effects of physical activity on appetite (i.e., such as bicycle ergometer, treadmill, swimming, and so on), since it had been reported that the hand-grip activity for 10 min can cause significant increase of the level of physical fatigue sensation and that this increase of the fatigue sensation can be classically conditioned^19,21^, we thought that the fatigue sensation caused by the hand-grip-activity for 10 min can be useful for assessing whether fatigue sensation has effects on appetite or not. Since we evaluated the alteration of the level of appetite caused by the hand-grip-activity during the conditioning session, whether the alteration of the level of appetite is caused by the low intensity activity such as hand-grip-activity can also be determined in our present study: In fact, as described in the Discussion, there have been no reports that assessed the alterations of the level of appetite-regulating hormones caused by performing hand-grip activity. We examined neural activity caused by viewing images of food in the presence of the fatigue sensation: It has been reported that examining neural responses to visual food cues is an effective approach to investigate the neural mechanisms of food-related behaviors^22^. We used magnetoencephalography (MEG) with high temporal and spatial resolutions to assess the neural activity caused by viewing images of food^23,24^ and performed spatial filtering analyses of the MEG data to obtain the data regarding the changes in oscillatory power reflecting changes in neural dynamics^25–27^.

## Methods

### Participants

Twenty healthy male volunteers aged 22.9 ± 1.9 years (mean ± standard deviation [SD]) participated in this study: Each participant was asked whether he had present illness, symptoms, and/or health problems by the form of questionnaire. None of the participants were underweight or obese. The participants’ mean body mass index was 21.7 ± 2.0 kg/m^2^. The participants were confirmed to be right-handed according to the Edinburgh Handedness Inventory^28^. Individuals taking chronic medications which affect the central nervous system and current smokers were excluded. Individuals with a history of brain injury, mental illness, or disorder of upper extremity were also excluded. The participants were required to refrain from caffeine for 12 h before each visit. The study protocol was approved by the Ethics Committee of Osaka City University (Approval No. 3769). Each participant provided written informed consent to participate in our present study in accordance with the principles of the Declaration of Helsinki and the Ethical Guidelines for Medical and Health Research Involving Human Subjects in Japan (Ministry of Education, Culture, Sports, Science, and Technology, and Ministry of Health, Labour and Welfare).

### Experimental design

**Figure 1:**
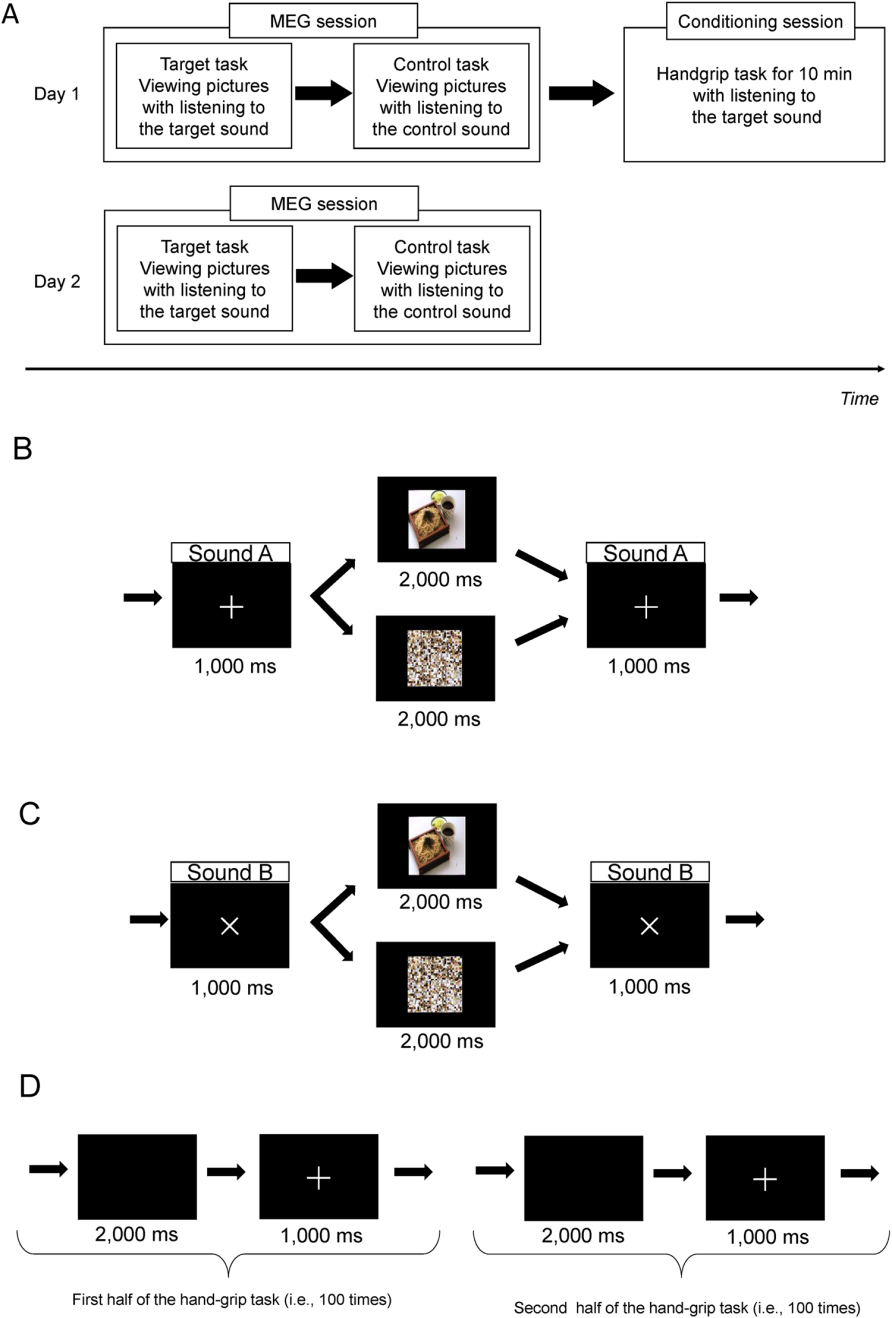
Experimental design. (**A**) The present study was performed on 2 consecutive days. The experiment on the first day (day 1) consisted of an magnetoencephalography (MEG) session and a conditioning session, and that on the second day (day 2) consisted of an MEG session that was identical to that on day 1. The MEG session consisted of a target task and a control task. Subjective levels of appetite (i.e., desire to eat), appetite for fatty foods, and fatigue sensation were assessed using a visual analog scale, just before and after the target and control tasks and conditioning session. In the target and control tasks, the participants were asked to view a visual stimulus consisting of food and mosaic images projected on a screen. (**B**) The visual presentation used in the target task consisted of a presentation of a target mark (the target mark was “+” in Fig. 1) for 1,000 ms followed by food and mosaic pictures for 2,000 ms; this sequence of visual presentations was played 260 times. (**C**) The visual presentation used in the control task consisted of a presentation of a control mark (the control mark was “×” in Fig. 1) for 1,000 ms followed by the food and mosaic images for 2,000 ms; this sequence of visual presentations was played 260 times. (**D**) In the conditioning session, the participants were instructed to grasp a hand-grip device in time with the visual cue. The visual cue consisted of a repetition of a blank screen for 2,000 ms followed by the target mark for 1,000 ms; the participants were asked to grasp the hand-grip device over the presentation of the target mark. For the last 100 times of the hand-grip trials, the target sound was played in time with the presentation of the target mark. The permission to include the food image in this figure was obtained from Kagawa Nutrition University Publishing Division.

The present study was performed on 2 consecutive days (Fig. 1A). On each day, all participants fasted overnight from 9:00 pm on the previous day (they were allowed to drink water) and were instructed to avoid intense exercise and to maintain normal sleeping hours^29^. The experiment on the first day (day 1) consisted of an MEG session and a conditioning session, and that on the second day (day 2) consisted of an MEG session that was identical to that on day 1. At the beginning of the experiment on day 1, the participants were asked to answer the Three-Factor Eating Questionnaire (TFEQ) to assess their normal eating behavior^30–32^. In the MEG session, the participants lay in a supine position on a bed in a magnetically shielded room. The MEG session consisted of two tasks: a target task and a control task. In the target and control tasks, the participants were asked to view a visual stimulus consisting of food and mosaic images projected on a screen placed in front of them using a projector (PG-B10S; Sharp, Osaka, Japan). The visual presentation used in the target task consisted of the presentation of a target mark (the target mark was “+” or “×”, as described below) for 1,000 ms followed by the food and mosaic images for 2,000 ms; this visual presentation sequence was played 260 times (Fig. 1B). The visual presentation used in the control task consisted of the presentation of a control mark (the control mark was “×” or “+”, as described below) for 1,000 ms followed by the food and mosaic images for 2,000 ms; this visual presentation sequence was also played 260 times (Fig. 1C). For the target and control tasks, half of the images were food and the other half were mosaics; these were presented in random order. We used typical Japanese food items as food images and the mosaic images were created from the food images as control images corresponding with food images using commercial software (Adobe Photoshop Elements 6.6, adobe systems Inc., San Jose, CA)^24,29^. The set of food images used in our present study was identical to that used in our previous studies^24,29^: Twenty pictures of typical modern Japanese food items, including steak, croquette, hamburger, fritter, chicken nugget, French fry, pizza, spaghetti, ice cream, fried dumpling, and fried rice, on Standard Tables of Food Composition in Japan^33,34^ were used in our present study. The participants were instructed to have appetitive motives for each food item, not to recall past experiences, and not to imagine the taste of each food item when the food images were presented^29^. In addition to the presentation of images, a target sound was played over the presentation of the target mark in the target task and a control sound was played over the presentation of the control mark in the control task. For half of the participants, the target mark was “+” and the target sound was an 8,000 Hz beep for 1,000 ms, and the control mark was “×” and the control sound was a 200 Hz beep for 1,000 ms. For the other half of the participants, the target mark was “×” and the target sound was a 200 Hz beep for 1,000 ms, and the control mark was “+” and the control sound was an 8,000 Hz beep for 1,000 ms. The target and control tasks were performed in random order. There was a rest session for 5 min between the target and control tasks. Neural activities during the target and control tasks were recorded using MEG.

On day 1, the participants performed a conditioning session after the MEG session. In the conditioning session, they were asked to perform hand-grip trials 200 times with their right hand using a hand-grip device (EXG006 30 kg; Alinco, Osaka, Japan). In these trials, they were instructed to grasp the hand-grip device in time with a visual cue (Fig. 1D). The visual cue consisted of a repetition of a blank screen for 2,000 ms followed by the target mark for 1,000 ms; the participants were asked to grasp the hand-grip device over the presentation of the target mark. For the last 100 times of the hand-grip trials, the target sound was played in time with the presentation of the target mark.

On day 2, the participants were asked to perform an MEG session identical to that on day 1. The experiments for three participants were performed between 9:30 am and noon, and those for the other participants were performed between 2:00 pm and 5:00 pm.

Subjective levels of appetite (i.e., desire to eat), appetite for fatty foods, and fatigue sensation were assessed using a visual analog scale (VAS) that ranged from 0 (minimum) to 100 (maximum) just before and after the target and control tasks and conditioning session^35^.

### MEG recording and analyses

The recording of MEG and magnetic resonance (MR) image and the analyses of these data were performed by a similar method to that of our previous study^36^. As originally described in^36^, MEG was recorded using a whole-head-type 160-channel MEG system (MEG Vision; Yokogawa Electric Corporation, Tokyo, Japan). The magnetic field resolution of the MEG system was 4 fT/Hz^1/2^ in the white noise region. The sensor and reference coils were gradiometers (15.5 mm diameter and 50 mm baseline), and the two coils were separated by 23 mm. The sampling rate was 1,000 Hz and the obtained data were high-pass filtered at 0.3 Hz.

As originally described in^36^, the preprocessing of the MEG data was performed as follows: The magnetic noise from outside the magnetically shielded room was eliminated by subtracting the data obtained from reference coils using specialized software (MEG 160; Yokogawa Electric Corporation) and epochs of the MEG data including artifacts were identified visually and excluded before the analyses. Spatial filtering analysis of the MEG data was performed to estimate changes in oscillatory brain activity reflecting time-locked cortical activities caused by performing the target and control tasks on day 2. The MEG data were band-pass filtered at 4–8 Hz, 8–13 Hz, and 13–25 Hz using a method of finite impulse response filtering implemented in Brain Rhythmic Analysis for MEG software (BRAM; Yokogawa Electric Corporation). The location and intensity of the cortical activities were estimated with BRAM after the band-pass filtering, which uses a narrow-band adaptive spatial filtering algorithm. Voxel size was set at 5.0 × 5.0 × 5.0 mm. For each MEG measurement (i.e., both the target and control tasks) and frequency band, the oscillatory power of the MEG data in the time window of 500 ms from 0 to 1,500 ms after the onset of the image presentation was calculated relative to that in the time period from −500 to 0 ms from the onset of the image presentation (i.e., baseline).

The MEG data analyzed with the spatial filtering method as described above were further analyzed using statistical parametric mapping (SPM8; Wellcome Department of Cognitive Neurology, London, UK), implemented in Matlab (Mathworks, Natick, MA). Normalization and smoothing of the MEG data was performed as described in^36^: The MEG parameters were transformed into the Montreal Neurological Institute T1-weighed imaged template and applied to the MEG data. The anatomically normalized MEG data were filtered with a Gaussian Kernel of 20 mm (full-width at half-maximum) in the x-, y-, and z-axes. Individual data were incorporated into a random-effects model and the parameters estimated were used to create “contrast” images for group analyses. Individual contrast images were used in a flexible factorial design with images (i.e., food or mosaic images) and tasks (i.e., conditioning or control tasks) as within-subject factors, and the interaction corresponding to the contrast between “food (control) – mosaic (control)” and “food (target) – mosaic (target)” was calculated for each frequency band and time window. The significance of the interaction was assessed on a voxel-by-voxel basis. The threshold for the analysis was set at *P* < 0.00278 (family-wise-error corrected for multiple comparisons), considering the multiple comparisons among frequencies, time windows, and the increase or decrease compared with baseline (i.e., three frequency bands × three time windows × increase or decrease = 18 comparisons). However, the statistical threshold of the statistical parametric map shown in our figure was set at *P* < 0.05 (family-wise-error corrected for multiple comparisons) for the purpose of presentation. For the brain regions that survived the statistical threshold in the flexible factorial design analysis, the contrast of the alteration in oscillatory power corresponding to {[food (control) – mosaic (control)] – [food (conditioning) – mosaic (conditioning)]} was calculated, and then used to assess the relationships between the alterations in oscillatory brain activity and those in subjective rating scores, such as appetite for fatty foods. Localization of the brain regions was performed using WFU_PickAtras, Version 3.0.4 (http://fmri.wfubmc.edu/software/pickatlas), Talairach Client (Version 2.4.3; http://www.talairach.org/client.html), and SPM Anatomy Toolbox^37^.

### MR image overlay

As originally described in^36^, to perform registration of magnetic source locations with their respective anatomical locations, anatomical MR image was obtained by using a Philips Achieva 3.0 TX (Royal Philips Electronics, Eindhoven, The Netherlands). Five markers (Medtronic Surgical Navigation Technologies, Inc., Broomfield, CO) were attached to the scalp (i.e., two markers 10 mm in front of the left and right tragus, one marker 35 mm above the nasion, and two markers 40 mm to either side of the marker above the nasion). The MEG data were overlaid on MR images using information obtained from the markers and MEG localization coils.

### Statistical analyses

Values are presented as mean ± SD unless otherwise stated. A paired *t*-test was used to compare the level of fatigue sensation, appetite, and hand-grip strength between before and after the target, control, and hand-grip tasks. Pearson’s correlation analysis was performed to assess the association between the neural activity induced by the target and/or control tasks on day 2 and the level of appetite assessed after the conditioning task on day 2. All *P* values were two-tailed, and values < 0.05 were considered statistically significant. All statistical analyses were performed using the IBM SPSS 21.0 software package (IBM, Armonk, NY).

## Results

### Fatigue sensation

**Figure 2:**
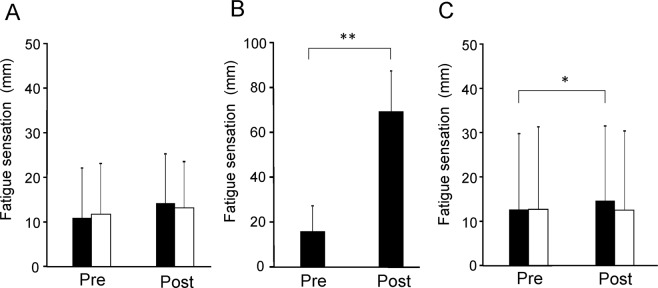
Subjective level of fatigue sensation. (**A**) Subjective levels of fatigue sensation before (Pre) and after (Post) the target and control tasks on day 1. Closed columns indicate values assessed before and after the target tasks. Open columns indicate values assessed before and after the control tasks. (**B**) Subjective levels of fatigue sensation before (Pre) and after (Post) the conditioning sessions. (**C**) Subjective levels of fatigue sensation before (Pre) and after (Post) the target and control tasks on day 2. Closed columns indicate values assessed before and after the target task. Open columns indicate values assessed before and after the control task. The participants were asked to rate their level of fatigue sensation on a 100-mm visual analog scale ranging from 0 (minimum fatigue sensation) to 100 (maximum fatigue sensation). Data are presented as mean ± SD. *P < 0.05 and **P < 0.01, paired *t*-test.

The levels of fatigue sensation after the target and control tasks on day 1 were not altered compared with those before the tasks (*t*_19_ = 2.035, *P* = 0.056 and *t*_19_ = 0.899, *P* = 0.380, respectively, paired *t*-test; Fig. 2A). The level of fatigue sensation after the hand-grip task was increased compared with that before the task (*t*_19_ = 10.647, *P* < 0.001, paired *t*-test; Fig. 2B). The level of fatigue sensation after the target task on day 2 was increased compared with that before the task (*t*_19_ = 2.230, *P* = 0.038, paired *t*-test; Fig. 2C). The level of fatigue sensation after the control task on day 2 was not altered compared with that before the task (*t*_19_ = 0.000, *P* = 1.000, paired *t*-test).

### The scores for the eating behavior

The scores for the eating behavior were assessed by TFEQ. The score for cognitive restraint was 12.2 ± 4.0, that for uncontrolled eating was 20.2 ± 4.7, and that for emotional eating was 9.7 ± 3.3.

### Hand-grip strength

**Figure 3:**
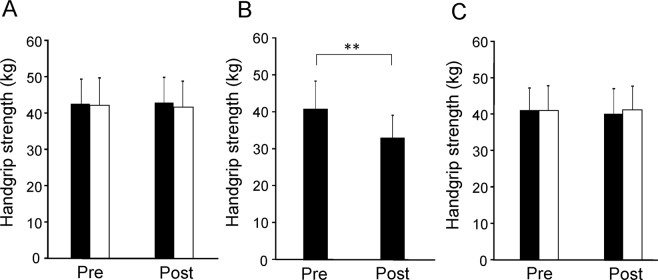
Grip strength. (**A**) Grip strength assessed before and after the target and control tasks on day 1. Closed columns indicate values assessed before (Pre) and after (Post) the target task. Open columns indicate values assessed before and after the control task. (**B**) Grip strength assessed before (Pre) and after (Post) the conditioning sessions. (**C**) Grip strength assessed before (Pre) and after (Post) the target and control tasks on day 2. Closed columns indicate values assessed before and after the target task. Open columns indicate values assessed before and after the control task. Data are presented as mean ± SD. **P < 0.01, paired *t*-test.

The levels of hand-grip strength after the target and control tasks on day 1 were not altered compared with those before the tasks (*t*_19_ = 0.201, *P* = 0.843 and *t*_19_ = 0.768, *P* = 0.452, respectively, paired *t*-test; Fig. 3A). The level of hand-grip strength after the hand-grip task was decreased compared with that before the hand-grip task (*t*_19_ = 9.041, *P* < 0.001, paired *t*-test; Fig. 3B). The levels of hand-grip strength after the target and control tasks on day 2 were not altered compared with those before the tasks (*t*_19_ = 1.331, *P* = 0.199 and *t*_19_ = 0.088, *P* = 0.931, respectively, paired *t*-test; Fig. 3C).

### Appetite

**Figure 4:**
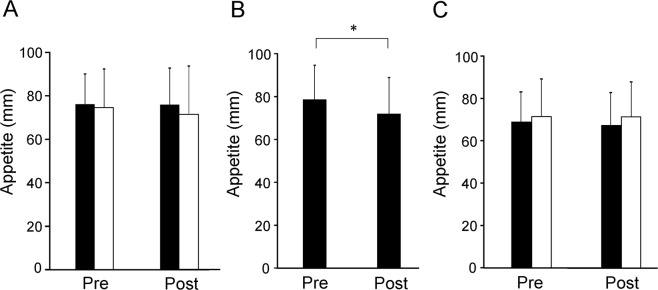
Subjective level of appetite. (**A**) Subjective levels of appetite before (Pre) and after (Post) the target and control tasks on day 1. Closed columns indicate values assessed before and after the target task. Open columns indicate values assessed before and after the control task. (**B**) Subjective levels of appetite before (Pre) and after (Post) the conditioning sessions. (**C**) Subjective levels of appetite before (Pre) and after (Post) the target and control tasks on day 2. Closed columns indicate values assessed before and after the target task. Open columns indicate values assessed before and after the control task. Participants were asked to rate their level of appetite on a 100-mm visual analog scale ranging from 0 (minimum appetite) to 100 (maximum appetite). Data are presented as mean ± SD. *P < 0.05, paired *t*-test.

The levels of appetite after the target and control tasks on day 1 were not altered compared with those before the tasks (*t*_19_ = 0.041, *P* = 0.968 and *t*_19_ = 0.545, *P* = 0.592, respectively, paired *t*-test; Fig. 4A). The level of appetite after the hand-grip task was decreased compared with that before the task (*t*_19_ = 2.589, *P* = 0.018, paired *t*-test; Fig. 4B). The levels of appetite after the target and control tasks on day 2 were not altered compared with those before the tasks on day 2 (*t*_19_ = 0.648, *P* = 0.525 and *t*_19_ = 0.000, *P* = 1.000, respectively, paired *t*-test; Fig. 4C). The level of appetite for fatty foods after the target task on day 2 showed a tendency toward increase compared with that before the task (*t*_19_ = 2.083, *P* = 0.051, paired *t*-test). The level of appetite for fatty foods after the control task on day 2 was not altered compared with that before the task (*t*_19_ = 0.536, *P* = 0.598, paired *t*-test). There were no correlations between the indices of the eating behaviors assessed by TFEQ and the alteration of the level of appetite for fatty food after the target task on day 2.

### Spatial filtering analyses of the MEG data

**Figure 5:**
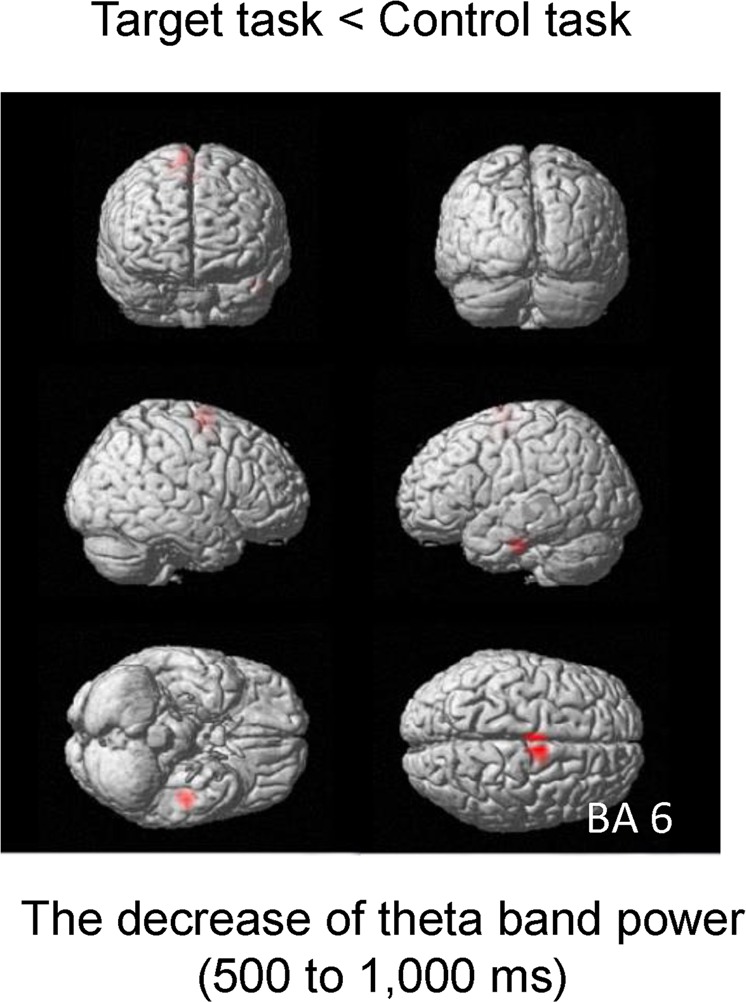
Statistical parametric map of the brain region where the level of the decreased theta band (4–8 Hz) power was lower in the control task than in the target task on day 2. Random-effect analysis of 14 participants, *P* < 0.05, family-wise-error corrected for the entire search volume.

**Figure 6:**
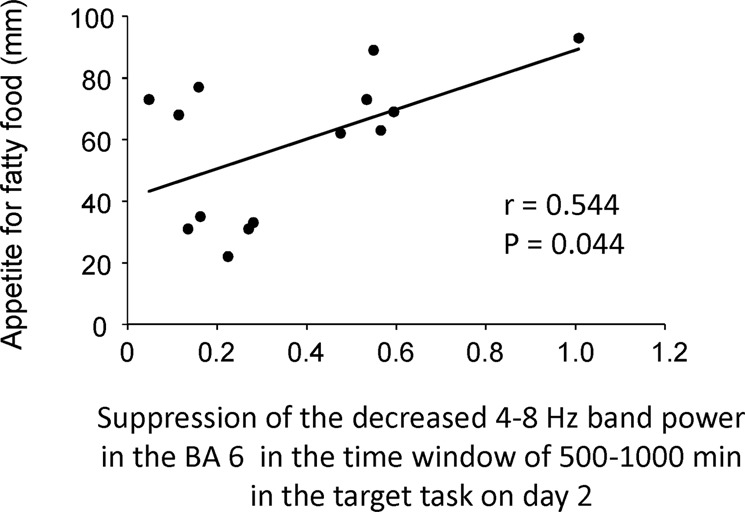
Relationship between the suppression of the decreased theta band (4–8 Hz) power in the right BA 6 in the target task on day 2 and the subjective level of appetite for fatty foods observed in the target task on day 2. The linear regression line, Pearson’s correlation coefficient, and the *P* value are shown.

Six participants were excluded from the MEG analysis because after the removal of artifacts, the numbers of epochs of their MEG data were not sufficient for analysis (i.e., MEG data with fewer than 40 epochs were excluded). To identify changes in neural activity caused by viewing the food images in the target task on day 2 compared with those caused by viewing the food images in the control task on day 2, the oscillatory brain activity observed during the target task on day 2 was compared with that observed in the control task on day 2. The decrease of theta band power in the right Brodmann’s area (BA) 6 in the time window of 500–1,000 ms after the onset of the image presentation observed in the target task on day 2 was smaller than that observed in the control task on day 2 (Fig. 5; Table 1). The suppression of the decreased theta band power in the right BA 6 in the target task on day 2 was positively associated with the increase of the level of appetite for fatty foods caused by performing the target task on day 2 (r = 0.544, *P* = 0.044; Fig. 6). There were no correlations between the indices of the eating behaviors assessed by TFEQ and the alteration in the oscillatory brain activity.

**Table 1:** Brain region showing a suppression of the decrease of theta band (4–8 Hz) power in the target task compared with that in the control task.

Frequency	Location	BA	MNI coordinate (mm)			Z value
			x	y	z	
4–8 Hz	Medial Frontal Gyrus	6	7	−2	60	4.64

BA, Brodmann area; MNI, Montreal Neurological Institute.

x, y, z: Stereotaxic coordinate.

Data were obtained from random-effect analysis. Only significant change is shown (paired *t*-test, *P* < 0.00278, family-wise error rate).

## Discussion

In the present study, the participants performed hand-grip trials while listening to a sound on day 1 and viewed food and mosaic images on day 2 while listening to the sound identical to that used on day 1. The hand-grip trials caused an increase in fatigue sensation and a decrease in hand-grip strength. On day 2, although listening to a sound caused an increase in fatigue sensation, no alteration in hand-grip strength was observed. The level of appetite for fatty foods showed a tendency toward increase after listening to the sound compared with that before listening to the sound on day 2. A suppression of the alteration of theta band power in BA 6 was observed when the participants viewed food images in the presence of fatigue sensation on day 2; this suppression was positively correlated with the level of appetite for fatty foods.

An increase in fatigue sensation was observed on day 2 only in the target task, thereby confirming that the association between fatigue sensation induced by hand-grip task trials and the sounds was learned during the conditioning session on day 1, and that the fatigue sensation was induced in the target task on day 2 without hand grip activity, as we had intended.

The neural activities caused by viewing food images in the presence of fatigue sensation without hand grip activity on day 2 were recorded using MEG, and alterations in oscillatory brain activity were assessed. Several studies have reported that alterations in oscillatory brain activity in the theta (4–8 Hz), alpha (8–13 Hz), and beta (13–25 Hz) bands are related to the neural mechanisms of appetite and/or fatigue. It has also been reported that increases in beta band power in the supplementary motor area (SMA) and decreases in theta band power in the dorsolateral prefrontal cortex are related to the suppression of motivation to eat^23^, that increases in alpha band power in the insular cortex are associated with the level of cognitive restraint of food intake^24^, and that decreases in theta band power in the posterior cingulate cortex and decreased alpha and beta band powers in the frontal cortex are related to the decision to rest in the presence of fatigue^38^. Therefore, we examined alterations in oscillatory brain activity in theta, alpha, and beta band powers caused by viewing food images in the presence of fatigue sensation without hand grip activity. The extent of the decrease in theta band power in the right BA 6 caused by viewing food images was suppressed in the presence of fatigue sensation (i.e., in the target task on day 2), and the level of suppression was positively associated with the level of appetite for fatty foods in the target task on day 2, suggesting that the right SMA is related to the increase of appetite for fatty foods observed in the target task on day 2.

The SMA has been reported to be involved in not only motor-related functions such as assembly of motor programs^39,40^, but also in neural mechanisms related to appetite^23^. An alteration of beta band power in the left SMA was observed when the participants were asked to suppress their motivation to eat, suggesting that the SMA plays a role in suppressing appetite. Furthermore, theta band oscillatory brain activity has been reported to be related to memory functions^41,42^. Taking these findings into consideration, one possible interpretation of our present results is that the decrease in theta band power in the SMA played a role in the suppression of appetite for fatty food through information processing related to past experiences and therefore, the suppression of the decrease in theta band power in the SMA observed in the target task on day 2 may be the reason for the increase of appetite for fatty food in the target task on day 2; however, it is difficult to determine causal relationships. Although our participants were instructed not to recall past experiences when the food images were presented, there may be a possibility that the information related to the past experiences was automatically (i.e., unintentionally) induced by viewing the food pictures. The alteration of theta band power was related to the suppression of appetite for fatty foods in the SMA in our present study while the alteration of beta band power in the SMA was related to the suppression of motivation to eat in the previous study^23^. This difference in the frequency bands (i.e., theta or beta bands) may be due to the difference in the ways the participants viewed the food images between the present and previous studies: The participants in our present study were not instructed to suppress appetite intentionally when they viewed the food images while the participants in the previous study was instructed to suppress their motivation to eat intentionally when they viewed the food images (i.e., the neural activity related to the suppression of appetite observed in our present study seems to reflect unintentionally activated neural processes rather than the intentionally induced neural processes observed in the previous study). The possible interpretation of the role of beta band brain activity in the suppression of appetite discussed above seems to be in line with the reports that the cortical oscillatory activity in beta frequency band is related to the movement inhibition observed in the Go/NoGo experimental paradigm (Picazio *et al*., 2014; Zhang *et al*., 2008) in that beta band oscillatory brain activity is involved in the intentional inhibitory control; however, the accumulation of findings regarding the functional roles of oscillatory brain activity in the neural mechanisms of appetite will be necessary to confirm our proposal. In addition, the difference between the role of the right and left SMA on appetite remains unclear; thus, further studies are needed on these points.

Several studies have examined the effect of physical activity on neural mechanisms related to appetite. In several functional magnetic resonance imaging (fMRI) studies, acute exercise on a cycle ergometer for 60 min was found to reduce neural responses to visual food cues in the insular and orbitofrontal cortices^43^, chronic exercise was observed to reduce neural responses to visual food cues in the insular cortex^44^, and physical exercise was found to decrease neural responses to high-calorie food images in the medial orbitofrontal and insula cortices^45^. Since alterations in neural activity in the brain regions related to food rewards, such as the insular and orbitofrontal cortices, were not observed in the present study, and the neural activity in the SMA was related to the increase of appetite induced by hand grip activity, there is a possibility that the regulation of appetite through fatigue sensation is based on the neural substrates different from that through appetite-regulating hormones.

In several other MEG studies, it was been reported that the presentation of visual food cues caused an equivalent current dipole in the insular cortex approximately 300 ms after the onset of the presentation of food cues^29^, and that neural activity related to the regulation of appetite in the dorsolateral prefrontal cortex, in this case, the suppression of appetite, was observed in the time window of 500–600 ms after the onset of the presentation of food images^23^. The fact that the alteration in theta band power in the SMA observed in the present study, which seems to be related the suppression of appetite, was in the time window of 500–1,000 ms after the onset of the image presentation is therefore in line with the previous MEG study.

Although appetite was not altered in the target task on day 2, appetite for fatty foods showed a tendency toward increase in the presence of fatigue sensation without hand grip activity. Some studies have reported that physical activity reduces appetite, as described in the Introduction^10–12^. In line with these previous reports, the level of appetite was decreased by performing hand-grip trials on day 1 in the present study; however, appetite for fatty foods was increased when fatigue sensation was induced without hand grip activity. Our findings suggest that fatigue sensation itself can increase appetite, especially that for fatty foods, when it is separated from physical activity. It has been proposed that an energy deficiency in the central nervous system is related to the pathophysiology of fatigue^46^. Taking this into consideration, it can be postulated that the increase of appetite for fatty foods in the fatiguing situation may be beneficial to the prevention and/or recovery from fatigue; however, since the increase of appetite for carbohydrates may also be beneficial to the prevention and/or recovery from fatigue, further study is needed on this point.

It is of great interest that fatigue sensation itself has the effect to increase appetite while physical activity reportedly suppresses appetite through the increased secretion of appetite-suppressing hormones^13–15^. Since hand-grip trials were not performed on day 2, it is plausible that the alterations of the level of appetite-regulating hormones were not caused by the target task on day 2 and the increase of appetite for fatty food observed in our present study may not be caused through the alterations in the level of appetite hormones.

The level of physical activity (i.e., hand grip activity) in our present study seem to be low compared with that of high intensity aerobic exercises for 60–90 mins such that reported to cause alterations in the level of appetite hormones and the suppression of appetite^13–15^. Although there have been no reports that assessed the alteration of the level of appetite hormones caused by hand grip activity, it has been reported that the increase of PYY plasma level caused by exercise is dependent on the intensity of the exercise^47^ and that the exercises below the aerobic-anaerobic threshold increase ghrelin level^48,49^, suggesting that the exercise-induced secretion of appetite hormones seems to be dependent on the intensity of exercise^50^. In fact, the level of appetite after the hand-grip task was decreased compared with that before the task in our present study (Fig. 4B); however, whether this alteration in appetite was due to the alterations of the level of appetite hormones or not is not clear in our present study.

There are limitations to the present study. First, the participants were all healthy males. To generalize our findings, studies with females, athletes, and obese individuals are needed. Second, the alterations of the level of appetite-regulating hormones caused by the conditioning sessions in the target and control tasks on day 2 were not assessed in the present study. Third, as only hand-grip trials were used in the present study, it is of great interest whether fatigue sensation caused by other types of physical activity, such as bicycle ergometers, and that caused by mental activity, can also increase appetite. Fourth, the experiments for three participants were performed between 9:30 AM and noon, and those for the other participants were performed between 2:00 PM and 5:00 PM in our present study. It is of interest whether the levels of appetite and fatigue sensation were influenced by the start time of our experiments. However, since the number of participants whose experiments were performed between 9:30 AM and noon was small, it is difficult to assess the effect of the start time of our experiments on the levels of appetite and fatigue sensation adequately in our present study. Fifth, we used the mosaic images as the control images in our present study to control for luminance, color, and local features. On the other hand, we cannot exclude the possibility that the activity of the object perception system in the target task was altered by the fatigue sensation compared with that in the control task and that the alteration in oscillatory brain activity in the SMA observed in our present study included neural activity related to the perception of objects at least in part. However, since the alteration in oscillatory brain activity in the SMA observed in our present study was related to the increase of appetite for fatty food, it is plausible that the oscillatory neural activity in the SMA observed in our present study was related to the processing of information regarding food and/or appetite rather than the processing of the features of objects that are not specific to food items.

In conclusion, the results of the present study suggest that fatigue sensation induced by hand grip activity may increase the appetite for fatty foods, and that this increase may be related to neural activity in the SMA, thereby suggesting that the SMA is related to the regulation of appetite for fatty foods. These findings are expected to contribute to gaining a better understanding of the neural mechanisms underlying the regulation of appetite in relation to fatigue.
